# Managing Hypercholesterolemia in Adults Older Than 75 years Without a History of Atherosclerotic Cardiovascular Disease: An Expert Clinical Consensus From the National Lipid Association and the American Geriatrics Society

**DOI:** 10.1111/jgs.19398

**Published:** 2025-04-10

**Authors:** Vera Bittner, Sunny A. Linnebur, Dave L. Dixon, Daniel E. Forman, Ariel R. Green, Terry A. Jacobson, Ariela R. Orkaby, Joseph J. Saseen, Salim S. Virani

**Affiliations:** ^1^ Division of Cardiovascular Disease University of Alabama at Birmingham Birmingham Alabama USA; ^2^ University of Colorado Skaggs School of Pharmacy and Pharmaceutical Sciences Aurora Colorado USA; ^3^ Department of Pharmacotherapy & Outcomes Science Virginia Commonwealth University School of Pharmacy Richmond Virginia USA; ^4^ Department of Medicine (Divisions of Geriatrics and Cardiology) University of Pittsburgh and Pittsburgh Geriatrics, Research, Education, and Clinical Center (GRECC), VA Pittsburgh Healthcare System Pittsburgh Pennsylvania USA; ^5^ Division of Geriatric Medicine and Gerontology Johns Hopkins University School of Medicine Baltimore Maryland USA; ^6^ Lipid Clinic and Cardiovascular Risk Reduction Program, Department of Medicine Emory University Atlanta Georgia USA; ^7^ New England Geriatric Education, Research and Clinical Center (GRECC), VA Boston Health Care System, Division of Aging Brigham & Women's Hospital, Harvard Medical School USA; ^8^ Department of Clinical Pharmacy and Department of Family Medicine University of Colorado Anschutz Medical Center Aurora Colorado USA; ^9^ Section of Cardiology, Department of Medicine The Aga Khan University Karachi Pakistan; ^10^ Texas Heart Institute and Baylor College of Medicine Houston Texas USA

**Keywords:** hypercholesterolemia, primary prevention, older adult

## Abstract

The risk of atherosclerotic cardiovascular disease increases with advancing age. Elevated LDL‐cholesterol and non‐HDL‐cholesterol levels remain predictive of incident atherosclerotic cardiovascular events among individuals older than 75 years. Risk prediction among older individuals is less certain because most current risk calculators lack specificity in those older than 75 years and do not adjust for co‐morbidities, functional status, frailty, and cognition which significantly impact prognosis in this age group. Data on the benefits and risks of lowering LDL‐cholesterol with statins in older patients without atherosclerotic cardiovascular disease are also limited since most primary prevention trials have included mostly younger patients. Available data suggest that statin therapy in older primary prevention patients may reduce atherosclerotic cardiovascular events and that benefits from lipid‐lowering with statins outweigh potential risks such as statin‐associated muscle symptoms and incident Type 2 diabetes mellitus. While some evidence suggests the possibility that statins may be associated with incident cognitive impairment in older adults, a preponderance of literature indicates neutral or even protective statin‐related cognitive effects. Shared decision‐making which is recommended for all patients when considering statin therapy is particularly important in older patients. Randomized clinical trial data evaluating the use of non‐statin lipid‐lowering therapy in older patients are sparse. Deprescribing of lipid‐lowering agents may be appropriate for select patients older than 75 years with life‐limiting diseases. Finally, a patient‐centered approach should be taken when considering primary prevention strategies for older adults.

**Abbreviations**
AGS: American Geriatrics Society
ASCVD: Atherosclerotic cardiovascular disease
CAC: Coronary artery calcium
CI: confidence interval
T2DM: Type 2 Diabetes mellitus
HR: Hazard ratio
LDL or LDL‐C: Low density lipoprotein (− cholesterol)
MI: myocardial infarction
NLA: National Lipid Association
NNT: Number needed to treat
non‐HDL‐C: non‐HDL‐cholesterol
OR: Odds ratio
PCE: Pooled cohort equations
SAMS: Statin associated muscle symptoms
U.S.: United States

## Preamble

Since 2014, the National Lipid Association (NLA) has issued several scientific statements on key aspects of the management of lipids and lipoproteins to prevent cardiovascular disease (https://www.lipid.org/practicetools/documents). The current Expert Clinical Consensus, focused on the treatment of hypercholesterolemia among individuals older than 75 years without clinically manifest atherosclerotic cardio vascular disease (ASCVD), was developed as a collaboration between the NLA and the American Geriatrics Society (AGS). The governing bodies of both organizations appointed members with relevant expertise to the writing group which was jointly chaired by a representative of NLA (V. B.) and AGS (S.A.L.). The Expert Clinical Consensus was developed by a diverse group of clinical lipidologists, cardiologists, geriatricians, and pharmacists with cumulative expertise in clinical medicine, geriatrics, cardiology, endocrinology, pharmacology, clinical trials, epidemiology, health outcomes research, and public health.

**FIGURE 1:**
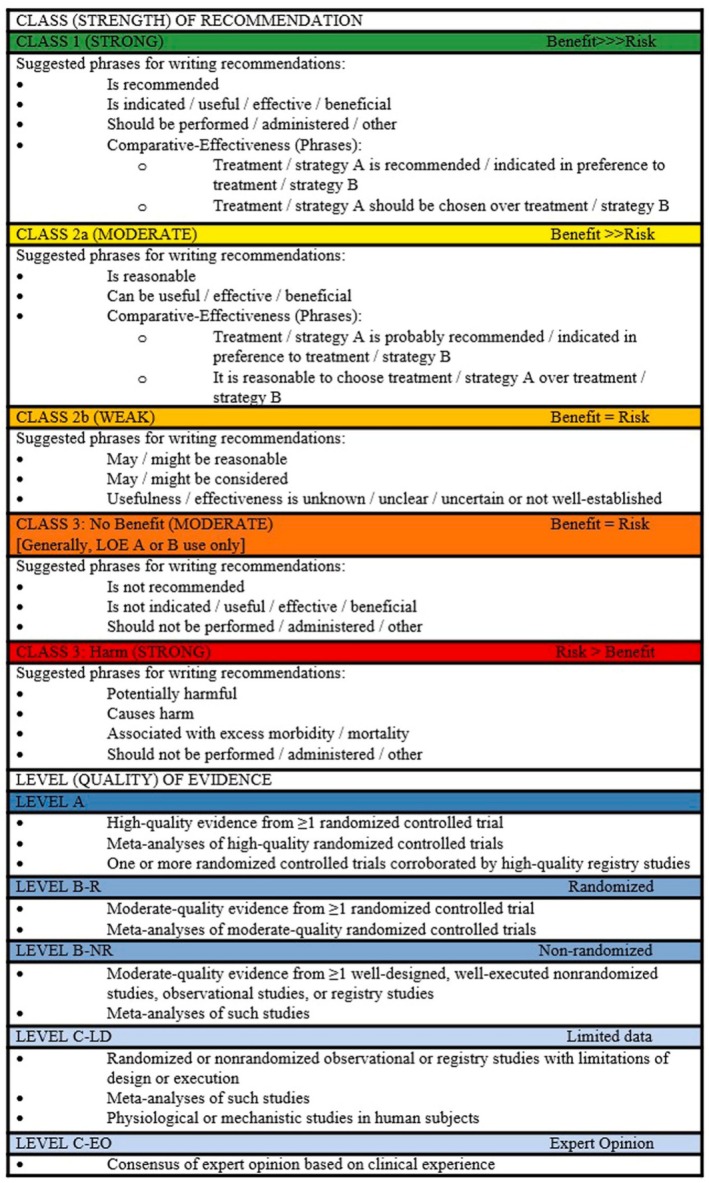
2019 Updated ACC/AHA Clinical Practice Guideline Recommendation Classification System (table modified from the 2019 ACC/AHA Clinical Practice Guideline Recommendation Classification System).^1^, ^2^

The chairs and members of the writing group jointly developed a set of key clinical questions to be addressed by the panel. Once the key clinical questions were agreed upon, writing assignments were jointly determined by the group based on content expertise with a primary and secondary author assigned to each question. The literature was reviewed and recommendations were developed using the 2019 Update of the American Heart Association (AHA) / American College of Cardiology (ACC) rating system for clinical guidelines, assigning a Class of Recommendation (I‐III) and a Level of Evidence (A‐C with sub categories) for each recommendation (Figure 1).^1^, ^2^ Each section and its associated recommendations were discussed in detail by the writing group. Preliminary recommendations were presented at the Annual Scientific Meeting of AGS (AGS23) in May 2023 and feedback from the audience was incorporated as appropriate. Final recommendations based on consensus of at least 60% of the expert panel were then reviewed by external peer reviewers, edited as appropriate and approved by the respective Boards of the NLA and AGS on 9/20/2024.

QUESTION 1 **For the population of adults older than 75 years without established ASCVD, what is the association between low‐density lipoprotein cholesterol (LDL‐C) and incident ASCVD?**

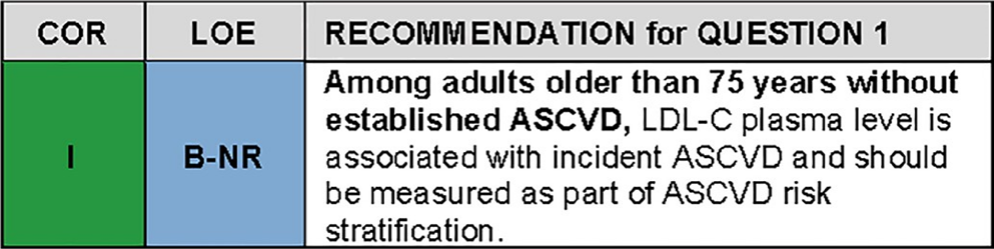

### Synopsis

Cross‐sectional and prospective cohort studies show that atherogenic lipoproteins increase with age from early adulthood to the 7th decade, but gradually decrease in subsequent decades.^3^, ^4^ This decrease among the oldest old (8th decade and beyond) is likely multifactorial including survivor bias (i.e., selective mortality at younger ages among those with the highest atherogenic lipoprotein levels) and declines in cholesterol associated with co‐morbidities (e.g., heart failure, malignancies, malnutrition). Many studies have shown strong and graded associations between LDL‐C level and incident ASCVD in women and men and across ethnicities among young and middle‐aged populations. In contrast, early epidemiologic analyses among older individuals suggested an inverse association between total cholesterol and outcomes which is most consistent with reverse causation.^5^ More contemporary analyses suggest that the association of LDL‐C level to incident ASCVD is maintained in the oldest age groups.^6^

### Recommendation‐specific supportive text

In 2007, a meta‐analysis of 61 prospective studies analyzed ischemic heart disease mortality as a function of total cholesterol or non‐high‐ density lipoprotein cholesterol (non‐HDL‐C) stratified by sex and age (5th to 9th decade).^7^ Relative risk of ischemic heart disease mortality related to the level of total cholesterol or non‐HDL‐C was highest among 40‐49 year‐olds. Relative risk decreased in a graded fashion with each advancing decade, but it remained statistically significant to age 80‐89 years. In contrast, absolute risk was lowest in the youngest age group and increased with advancing age.

**FIGURE 2:**
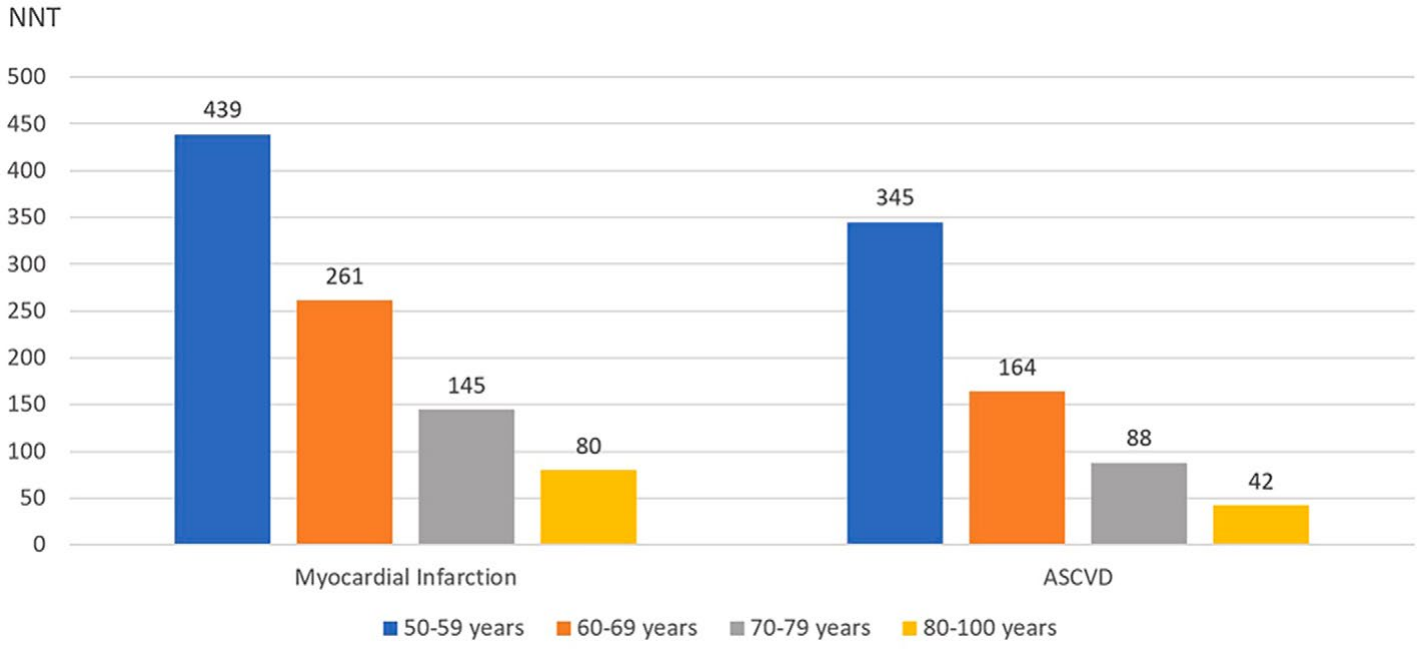
Number needed to treat for primary prevention of MI and ASCVD by age. Data from the Prospective Copenhagen General Study.^6^ The graph shows the number needed to treat (NNT) to prevent 1 event over 5 years for 1 mmol/L (~39 mg/dL) lower LDL‐C by age group for the endpoints myocardial infarction and ASCVD. NNT declines with advancing age consistent with greater benefit in the older compared to younger age groups.

The prospective Copenhagen General Study investigated the association of LDL‐C with incident myocardial infarction (MI) and ASCVD in 91,131 participants ranging in age from 20‐100 years old; 10,591 participants were 70‐79 years old and 3,188 participants were 80‐100 years old.^6^ Higher LDL‐C was predictive of incident MI and ASCVD independent of age. As in the Prospective Studies Collaboration, relative risks of MI and ASCVD (expressed as adjusted hazard ratios (HRadj)) were highest in the youngest (20‐49 year‐old) age group (MI HRadj 1.68 (95% CI 1.45‐1.87); ASCVD HRadj 1.47 (95% CI 1.32‐1.64)) and lowest in the oldest (80‐100 year‐old) age group (MI HRadj 1.28 (95% CI 1.08‐1.52); ASCVD HRadj 1.16 (95% CI 1.05‐1.29)). By contrast, absolute risk of events was lowest in the youngest age group and highest in the oldest age group. Based on these prospective observational data, the authors estimated the number needed to treat (NNT) to prevent MI or ASCVD for every 1 mmol/L (38.67 mg/dL) lowering of LDL‐C over 5 years. The estimated NNT declined with each decade of age (Figure 2). For ASCVD, the estimated NNT for 80‐100 year‐old participants was 42 compared to 345 for 50‐59 year‐old participants; for MI, the respective estimated NNTs were 80 and 439. Thus, LDL‐C is an important risk factor among patients older than 75 years and should be measured for ASCVD risk stratification.

QUESTION 2 **For the population of adults older than 75 years without established ASCVD, how should ASCVD risk be assessed and stratified?**

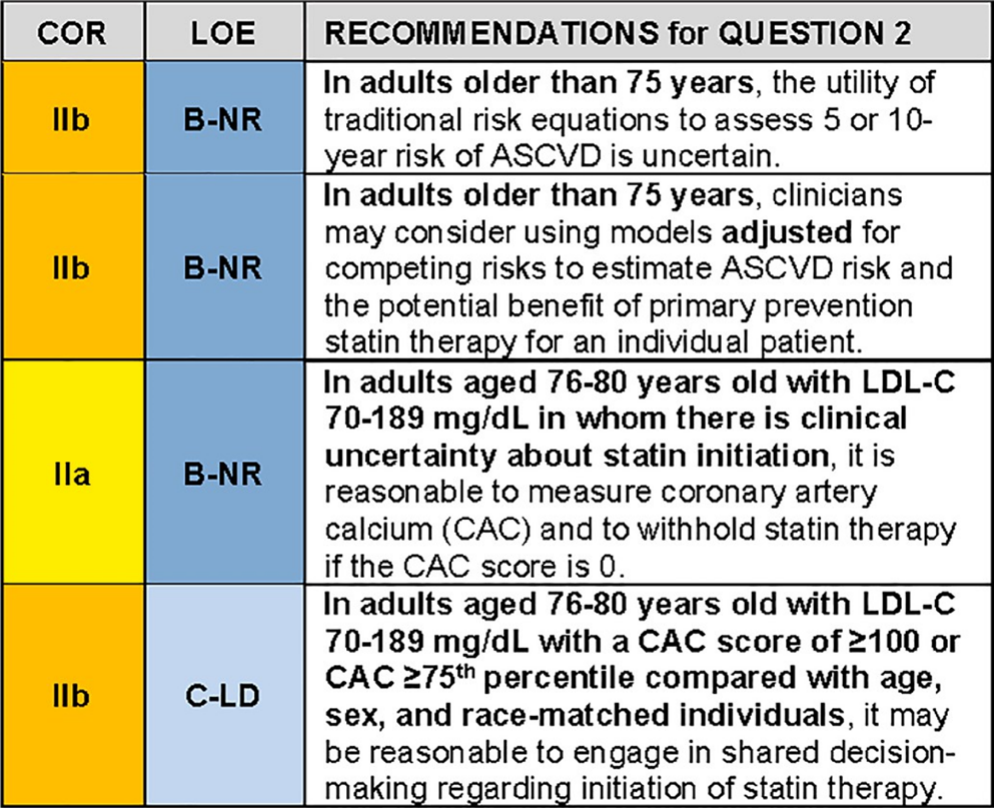

### Synopsis

Calibrating the use and intensity of preventive therapies to an individual's absolute ASCVD risk is the foundation of risk assessment.^8^, ^9^ Generally, this includes (a) the use of risk stratification tools to estimate 10‐year risk of an ASCVD event, (b) personalizing this risk estimate, and (c) then further refining this risk using imaging in select patients. For older adults, several risk stratification tools are available (Table 1). Since chronologic age is one of the most heavily weighted risk factors in all the currently available risk stratification tools, these tools are limited by a lack of specificity in identifying older adults most likely to benefit from preventive therapies including statin therapy. Most risk stratification tools do not incorporate other considerations that are very important in older adults including co‐morbidities, life expectancy, functional status, frailty, and cognition and most do not take into account the competing risk of non‐cardiovascular mortality^10^, which may lead to the over‐estimation of ASCVD risk and of the potential benefit from risk factor reduction. Use of models that adjust for competing risk in older adults can help estimate individualized treatment effects and inform clinical decision making. Assessment of vascular age using markers of subclinical atherosclerosis (e.g., CAC scoring [Table 2]) can assist clinicians in refining risk assessment and identifying older adults most likely to benefit from preventive therapies including statin therapy. Lifestyle therapy including a heart healthy diet and physical activity remains first line therapy in older adults, regardless of the results of risk assessment, given the independent association with all‐cause mortality and favorable impact on physical functioning and mental wellbeing.

**TABLE 1:** Major risk assessment algorithms available to assess ASCVD risk in older adults.

Risk Assessment Algorithm (Models not adjusted for competing risk)	Population	Outcomes/Comments
Pooled Cohort Risk Equations^11^ https://tools.acc.org/ascvd‐risk‐estimator‐plus/#!/calculate/estimate/ http://static.heart.org/riskcalc/app/index.html#!/baseline‐risk	Derived from 5 community‐based cohorts of Black and White participants in the U.S. Sex and race specific risk calculator for ASCVD risk assessment in 4 groups: white men, white women, black men, black, women.	Estimates 10‐year risk of hard ASCVD events (nonfatal MI, CHD death, fatal or nonfatal stroke). Variables used to calculate ASCVD risk include age, sex, race, total cholesterol, HDL‐C, SBP, antihypertensive therapy, history of diabetes mellitus, and current smoking. Reasonably well calibrated in the general U.S. population. Large numbers of studies available on reclassification by CAC. Available for non‐Hispanic White and non‐Hispanic African American patients 40–79 years of age. Small number of adults above age 70 (predominantly contributed by the Cardiovascular Health Study).
Reynolds Risk Score^12^, ^13^ http://www.reynoldsriskscore.org/	Score derived from healthcare professionals (mostly White patients) enrolled in clinical trials. Input variables include age, sex, total cholesterol, HDL‐C, SBP, current smoking, hsCRP, and parental history of MI before age 60 years.	Outcomes include CHD death, nonfatal MI, fatal or nonfatal stroke, and coronary revascularization. Uncertain utility in other ethnic groups and reclassification with CAC not well known.
Framingham General CVD Risk Score^14^ https://reference.medscape.com/calculator/252/framingham‐risk‐score‐2008 #	Developed in a predominantly White population of Framingham study participants. Utility in other ethnic groups is not known. Developed for individuals 30–74 years of age.	Assesses risk of total CVD (CHD death, MI, coronary insufficiency, angina, ischemic stroke, hemorrhagic stroke, transient ischemic attack, intermittent claudication, and heart failure. Input variables include age, sex, total cholesterol, HDL‐C, SBP, antihypertensive therapy, history of diabetes mellitus, and current smoking.
QRISK3^30^ https://qrisk.org/three/index.php	Risk score developed using a total of 1309 general practices in England: data from 981 practices were used to develop the scores and data from a separate set of 328 practices were used to validate the scores. 7.89 million patients aged 25–84 years were in the derivation cohort and 2.67 million patients were in the validation cohort. Mean age between 42–43 years in the derivation and validation cohorts.	Outcomes include CHD, ischemic stroke, or transient ischemic attack. Several traditional risk factors used, as in PCE, but also includes several additional variables including BMI, SBP variability, corticosteroid use, chronic kidney disease, atrial fibrillation, erectile dysfunction in men, migraine, rheumatoid arthritis, SLE, severe mental illness, HIV, atypical antipsychotic use, Townsend score (measure of material deprivation) etc. Applicability to broad U.S. population is not known. Impact of reclassification with CAC is unclear.
Models Adjusted For Competing Risk		
SCORE2‐OP risk prediction algorithms^10^ https://www.heartscore.org/en_GB	Risk model to estimate 5‐ and 10‐year risk of cardiovascular disease in individuals aged over 70 years in four geographical risk regions in Europe. Competing risk and sex‐adjusted.	Outcomes include non‐fatal myocardial infarction, non‐fatal stroke, and cardiovascular mortality. Secondary outcomes include hospitalization from heart failure. The external validation showed C index for discrimination ranging between 0.63 (95% CI 0.61–0.65) and 0.67 (95% CI 0.64–0.69). Although external validation cohorts included U.S. cohorts, the current risk charts are only available for use in 4 regions in Europe with unclear validation across various ethnic groups in the U.S. Impact of reclassification by CAC unclear.
NORRISK2^17^	Model adjusted for competing risk for estimating 10‐year risk of myocardial infarction or stroke. Developed using data from the prospective Cohort of Norway (CONOR) study linked to the Cardiovascular Disease in Norway (CVDNOR) project, a database of all hospital stays with a CVD‐related discharge diagnosis in Norway. Developed in 31,445 men and 35,267 women in 1994–1999. External validation population consisted of 19,980 men and 19,309 women in 2000–2003.	The resulting diagrams depict 10‐year risks of myocardial infarction or stroke by serum total cholesterol, systolic blood pressure and smoking status to help clinicians determine which patients meet recommended thresholds for statin and antihypertensive treatment. A web‐based scoring tool is being developed. Limitations of NORRISK2 are that it may have limited generalizability outside Norway, and the population only included men and women up to age 79. Figure depicting estimated 10‐year risk is available here: https://doi.org/10.1177/2047487317693949
PROSPER^18^	Derived using competing risk analysis for myocardial infarction, stroke and vascular death in patients 70 or older with (*N* = 2550) and without (*N* = 3253) vascular disease from the “PROspective Study of Pravastatin in Elderly at Risk” (PROSPER) trial. Validated in the “Secondary Manifestations of ARTerial disease” (SMART) cohort study (*N* = 1442) and the “Anglo‐Scandinavian Cardiac Outcomes Trial‐Lipid Lowering Arm” (ASCOT‐LLA) trial (*N* = 1893). Covariates included glomerular filtration rate and the number of medications per patient as a measure of comorbidity.	The model was fitted for the prediction of 3.2‐year risk (median follow‐up), and these estimates were extrapolated to derive 5‐year and 10‐year CVD event risks. By entering patient characteristics in the model formula, it is possible to estimate the CVD risk and absolute risk reduction (ARR) with and without statin treatment for an individual patient; however, an interactive clinical tool has not been developed. The ARR can be translated into an individual number needed to treat—the number of patients with the same risk profile who would need to be treated to prevent 1 event in 5 or 10 years. Limitations of this model are that it was derived and validated only in Northern European populations, and results cannot be extrapolated to the very old (≥85 years) and to patients with stage IV or V chronic kidney disease (eGFR <30 mL/min), since they were not enrolled in these studies. Formula for estimating risk and absolute risk reduction with and without statin treatment for an individual patient is available here: https://doi.org/10.1007/s00392‐016‐1023‐8
CHS‐Rotterdam Study Model^20^	Risk prediction model specific for CHD (nonfatal MI and coronary death) that accounts for competing risk of death from non‐coronary causes Derived from 2 observational cohort studies of individuals 65 years or older who were free of cardiovascular disease: Cardiovascular Health Study (CHS) *N* = 4946 and Rotterdam Study *N* = 4303 Median age in CHS was 72 years for men and 71 years for women; median age in the Rotterdam Study was 73 years for men and 76 years for women. Covariates included age, systolic blood pressure, in the Rotterdam Study.	The prediction model had moderate ability to discriminate between events and nonevents (c‐statistic, 0.63 in both U.S. and European men and 0.67 and 0.68 in U.S. and European women). The model was well‐calibrated (good agreement between predicted and observed risks). Note that the number of non‐coronary deaths exceeded the number of CHD events over the entire age range and this gap was more pronounced at older ages. May be less applicable to non‐white populations. There is no clinician‐accesible web version for this model.

Table Footnote: The utility of traditional risk equations to assess 5 or 10‐year risk of ASCVD in older patients is uncertain and clinicians should consider using the competing‐risk adjusted models.

**TABLE 2:** Key studies summarizing the role of CAC in risk stratification in older adults.

Study	Patient Characteristics	Outcomes/Results
MESA^21^	6814 participants 964 (14%) between 75–85 years of age Follow‐up of 11.1 years	CAC 0, 1–100, 101–300, >300 was noted among 19%, 30%, 20%, and 31% of the participants aged ≥75 years. Ten‐year ASCVD event rates of 5.6, 14.3, 18.1, and 24.7 among individuals with CAC 0, 1–100, 101–300, and > 300, respectively. In multivariable models, each doubling of CAC was associated with HR (95% CI) of 1.12 (1.06–1.17).
CAC Consortium^22^	44,052 individuals referred for CAC 1663 (3.8%) ≥ 75 years of age	Prevalence of CAC 0, 1–100, 101–400, and > 400 was 16%, 23%, 25%, 36% respectively among those aged ≥75 years. Estimated survival at a mean follow‐up of 5.6 years was 98.1%, 92.3%, 91.3%, and 81.1%, respectively among those with CAC 0, 1–100, 101–400, and > 400.
Rotterdam Study^23^	2028 asymptomatic participants Aged 69.6 ± 6.2 years Median follow‐up was 9.2 years	Median CAC score was 84 Agatston units (AU) (25th to 75th percentile: 8 to 382 AU). 10.5% with CAC 0 Individuals were classified into low (<10%), intermediate (10% to 20%), and high (>20%) 10‐year coronary risk categories based on a Framingham refitted risk model. HR for ln(CAC +1) = 1.33 (1.21–1.47) in the multivariable regression model. Addition of CAC improved the C statistic from 0.72 to 0.76. 52% of participants in the intermediate group were reclassified by CAC into either low‐risk or high‐risk groups; Net Reclassification Index (NRI) not available. CAC values above 615 or below 50 AU were found appropriate to reclassify persons into high or low risk, respectively.
BioImage Study^24^	5805 participants Median follow‐up = 2.7 year Mean age = 69 years	CAC 0, 1–99, and ≥ 100 were seen in 32%, 29%, and 39%, respectively. Carotid imaging was performed to detect and quantify carotid plaque burden (cPB). The top CAC group comprised those with CAC ≥100, and the top cPB group comprised those with cPB ≥300 mm2 (chosen to match the percentile for CAC ≥100, giving 2 top groups of similar size but selected differently). cPB was zero in 23%. 86% of participants were statin eligible (10‐year ASCVD risk ≥ 7.5%). HR for CVD events were 1.48 (0.75–2.92) and 3.98 ((2.20–7.18) for CAC 1–99, and CAC ≥100 respectively compared to CAC 0 (referent). For cPB, the HR for CVD events were 1.23 (0.67–2.26) and 2.14 (1.20–3.78) for cPB 1–299, and ≥ 300 mm^2^ compared with no cPB. For cardiovascular disease events (including revascularization), the NRI was 0.14 for CAC and 0.06 for CPB. The positive NRIs were driven primarily by down classifying the large subpopulation with CAC 0 or cPB 0.
Atherosclerosis Risk in Communities (ARIC) Study^25^	1545 participants without coronary artery disease, stroke, or heart failure (HF) aged 75–94 years Median follow‐up = 1.1 year	CAC score of 0–99, 100–299, 300–999, and ≥ 1000 were seen in 34,5%, 18.8%, 28%, and 18.6% of the participants, respectively. 10% with CAC 0. Low number of events (22 ASCVD events, and 16 HF events). When compared with CAC 0–99 (referent category), the risk of ASCVD and HF was 4.41 (1.37–14.49) and 4.11(1.06–15.90), respectively for those with CAC ≥1000. When CAC was modeled as a continuous variable, a graded dose response was seen for ASCVD risk. For HF, the risk gradient was flat when evaluating CAC scores <300 but the risk increased steeply above this level of CAC.

### Recommendation‐specific supportive text

1. Although several ASCVD and global cardiovascular disease risk stratification algorithms are available for clinicians to use in individuals older than 75 years (Table 1)^8^, ^10^, ^11^, ^12^, ^13^, ^14^, they have notable limitations including a small number of individuals in this age group in the derivation cohort, limited non‐white enrollment, or derivation in countries outside the United States, all limiting generalizability to the overall U.S. population.
  The pooled cohort risk equations (PCE)^11^, which are sex and race‐specific were derived from five community‐based cohorts of Black and White participants in the U.S. aged 40‐79 years and are generally considered most applicable to the U.S. population. Like other risk stratification algorithms, the PCE are also heavily influenced by age, yielding risk estimates among older individuals with favorable risk profiles that are well above the traditional 10‐year ASCVD risk thresholds used to perform risk discussion regarding statin treatment. Despite these limitations, the use of the PCE could be helpful in individual patients. First, clinicians could use the 10‐year risk estimation as a medium to communicate risk in a numeric form to the patient and to discuss healthy lifestyle choices with the patient.^15^ Second, a higher risk may identify which risk factor(s) might be driving the higher ASCVD risk in an older adult and allow clinicians and patients to focus prevention efforts on those specific risk factors (e.g., uncontrolled hypertension, smoking). Clinicians could discuss how the patient's 10‐year ASCVD risk would be reduced if all risk factors were optimal. This could motivate patients to improve their adherence to lifestyle therapy and preventive medications. Recently, the AHA published risk equations (Predicting Risk of CVD EVENTs [PREVENT])^16^ which assess the risk of cardiovascular events (ASCVD and heart failure). These equations included adults between the ages of 30‐79 years of age and adjusted for competing risk of non‐cardiovascular deaths. In addition to traditional risk factors included in PCEs, PREVENT also includes statin use and estimated glomerular filtration rate as predictors in the model. PREVENT equations also allow the use of optional predictors such as urine albumin to creatinine ratio, hemoglobin A1C, and social deprivation index. Although PREVENT includes heart failure as an outcome in addition to ASCVD, which is of importance especially for older adults, it remains to be seen if PREVENT performs better than PCE or other risk equations in individuals older than 75 years.^16^
2. There are 4 competing‐risk adjusted models for ASCVD risk estimation in older adults (SCORE2‐OP, NORRISK2, PROSPER, and the CHS‐Rotterdam model).^10^, ^17^, ^18^, ^19^, ^20^ Use of these models can help estimate individualized treatment effects and inform clinical decision making, though it should be noted that they are not yet standard of care. An interactive tool that uses one of these models, SCORE2‐OP, is available online (https://www.heartscore.org/en_GB). Limitations of the models are that they may have limited generalizability outside the Northern European populations in which they were developed, and results of NORRISK2 and PROSPER cannot be extrapolated to the very old (≥85 years). Lastly, some important determinants for ASCVD risk in older adults were not included in all the models, such as kidney function or other markers of frailty.
3. Several epidemiologic studies have shown that adults aged 75 years and older with CAC = 0 have low ASCVD event rates and mortality (Table 2).^21^, ^22^, ^23^, ^24^, ^25^ In the 10‐year follow‐up from the MESA study^21^, the 10‐year ASCVD rate was 5.6% among those aged 75 years or older with CAC = 0. In the CAC consortium^22^, individuals 75 years or older with zero CAC had a 5.6‐year survival rate of 98%. Clinicians should note that the prevalence of CAC = 0 among older adults varies in these studies (19% in MESA and 16% in CAC consortium among those 75 years or older versus 10.5% among participants in the Rotterdam Study [mean age 69.6, SD = 6.2 years]). This reflects the population studied (epidemiologic cohort versus a referred population) as well as the characteristics of the studied population (White and South Asian^26^ populations and males have higher proportion with CAC > 0 compared with other ethnic groups, and women). Given the very low ASCVD risk and overall mortality associated with CAC = 0 in these studies, it is reasonable to withhold statin therapy in older adults for primary ASCVD prevention, especially since CAC = 0 is associated with low ASCVD event rates in the short to intermediate term time horizon.^27^
4. Older adults with subclinical atherosclerosis, as identified by CAC score ≥ 100 have a higher risk of ASCVD events. In the MESA study^21^, CAC scores of 1–100, 101–300, and > 300 were noted in 30%, 20%, and 31% of individuals aged 75 years or older, respectively, with corresponding 10‐year ASCVD event rates of 14.3, 18.1, and 24.7%, respectively. Using a cut‐off score of 100, approximately 50% of in‐ dividuals older than 75 years will be identified as “high‐risk” providing an opportunity for a risk‐based discussion regarding statin therapy in such individuals. Prevalence of CAC≥100 varies based on sex and ethnicity with lower prevalence in women, Chinese, Black and Hispanic individuals compared with men, Whites, and South Asian individuals. Although absolute CAC scores generally predict ASCVD outcomes over a much shorter time‐horizon compared with percentiles which generally predict long‐term or lifetime ASCVD outcomes,^28^ it is reasonable to use age‐, sex‐, race‐based percentile cut‐offs in this age segment given high absolute CAC prevalence. An age, sex, race‐based CAC score ≥ 75th percentile may identify patients at high‐risk of short‐term ASCVD events^29^ (https://www.mesa‐nhlbi.org/Calcium/input.aspx) Therefore, in adults aged 76–80 years with LDL‐C 70–189 mg/dL with a CAC score of ≥100 or CAC ≥75th percentile compared with age, sex, and race matched individuals, it may be reasonable to engage in shared decision making regarding initiation of statin therapy even though randomized controlled trial (RCT) evidence that statin therapy reduces ASCVD risk in individuals identified as high risk using a CAC‐based approach is limited. The decision should incorporate patient values and preferences as well as consideration of significant competing health risks, cognitive and functional status, and life expectancy.

QUESTION 3 **In the population of adults older than 75 years without established ASCVD, does statin therapy reduce ASCVD events?**

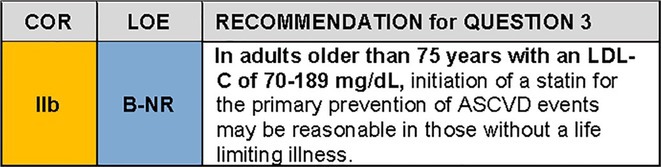

### Synopsis

Statin intensity refers to the percentage of expected lowering of LDL‐ C in the general adult population and is detailed in Table 3 which is adapted from the 2018 AHA/ACC Multisociety guidelines.^31^ Two nationwide studies from Denmark provide insights on LDL‐C lowering with statins and ASCVD event reduction among adults older than 75 years. Corn et al. showed that LDL‐C response to low to moderate‐intensity statins was slightly greater (2‐5% depending on statin and dose) among adults without ASCVD older than 75 years compared to individuals age 50 or younger.^32^ Differences in response to high‐intensity statin therapy were minimal. Andersson et al. compared the incidence of major vascular events (acute coronary syndrome, non‐hemorrhagic stroke and coronary revascularization) between individuals aged 50‐69 years with those aged 70‐74, 75‐79, 80‐84, and 85 years or older. Over a median follow‐up of 2.5 years, there were no differences by age group in major vascular event reduction per 1 mmol/L reduction in LDL‐C with adjusted HR of 0.76, 0.73, 0.81, 0.81, and 0.77, respectively.^33^

**TABLE 3:** Statin intensity as defined in the 2018 AHA/ACC/Multisociety guidelines.^31^

	Expected Mean LDL‐C Lowering*	Daily Dose (mg)
High‐Intensity	≥50%	Atorvastatin 40–80
		Rosuvastatin 20–40
Moderate‐Intensity	30 to 49%	Atorvastatin 10–20
		Rosuvastatin 5–10
		Simvastatin 20–40
		Pravastatin 40–80
		Lovastatin 40–80
		Fluvastatin 80
		Pitavastatin 1–4
Low‐Intensity	<30%	Simvastatin 10
		Pravastatin 10–20
		Lovastatin 20
		Fluvastatin 20–40

* Based on the total adult population (not exclusively patients older than 75 years).

Among individuals without ASCVD up to age 75 enrolled in randomized controlled outcomes trials of statin therapy, the average time to benefit was 2.5 years for reduction of ASCVD events.^34^ Whether this can be extrapolated to individuals older than 75 years is unknown. Of 186,854 participants in 28 major statin trials, only 8% were over age 75, and only 2% of those in primary prevention trials were over age 75.^35^ When data from these trials were analyzed in aggregate and trials among patients with heart failure or on dialysis were excluded, there was no statistical heterogeneity by age in the protective effect of statins for reduction of major cardiovascular events or vascular deaths per 1 mmol/L LDL‐C reduction over 4.9 years of follow‐up.^35^ In contrast, a trend towards smaller proportional risk reductions with advancing age was apparent among trial participants without a history of vascular disease.^35^

### Recommendation‐specific supportive text

To date, 6 statin trials have enrolled participants older than 75 years, with few individuals over 80 years.^36^ (Table 4) Two of these trials were mixed primary and secondary prevention (the Heart Protection Study (HPS)^37^ and Prospective Study of Pravastatin in the Elderly at Risk (PROSPER)^38^), while 3 had an upper age limit of 79‐82 years (HPS^37^, PROSPER^38^ and Anglo‐Scandinavian Cardiac Outcomes Trial—Lipid Lowering Arm (ASCOT‐LLA)^39^). PROSPER is notable as the only trial specifically conducted in older adults; however, data on the 2,355 participants over age 75 has only been published in aggregate, including those both with and without prior ASCVD.^38^

**TABLE 4:** Randomized controlled clinical trials in primary prevention among individuals 75 years or older.

Trial	Study characteristics	Intervention, Median Duration	Primary outcome and results	Older adult specific results
Heart Protection Study (HPS)^37^ 2002	‐ 20,536 adults, 40–80 years ‐ >75 years subgroup 1263 ‐ mixed primary and secondary prevention ‐ United Kingdom	Simvastatin 40 mg daily vs. placebo 5 years	Composite: deaths from all causes, from coronary heart disease, and from all other causes ‐ Significant reduction in all‐cause mortality: 1328 (12.9%) vs. 1507 (14.7%) events among statin vs. placebo, *p* = 0.0003	Absolute event rate for statin vs. placebo among those >75 years: 23.1% vs. 32.3%, *p* = 0.0002
Prospective Study of Pravastatin in the Elderly at Risk (PROSPER)^38^ 2002	‐ 5804 adults, 70–82 years, primary prevention subgroup *n* = 3239 ‐ >75 years in the overall trial, *n* = 2355 ‐ mixed primary and secondary prevention ‐ Ireland, Scotland, Netherlands	Pravastatin 40 mg daily vs. placebo 3.2 years	Composite: definite or suspected death from coronary heart disease, non‐fatal myocardial infarction, and fatal or non‐fatal Stroke ‐ Significant reduction in the primary endpoint: 408 vs. 473 events among statin vs. placebo, HR 0.85, 95% CI 0.74–0.97, *p* = 0.014	Primary prevention subgroup absolute event rate for statin vs. placebo (mean age 75): 11.4% vs. 12.1%, HR 0.94, 95% CI 0.77–1.15
Antihypertensive and Lipid‐Lowering Treatment to Prevent Heart Attack Trial—Lipid‐Lowering Trial (ALLHAT‐ LLT)^41^, ^46^ 2002	‐ 10,355 adults aged ≥55 years ‐ >75 years *n* = 1420—primary prevention ‐ North America	Pravastatin 40 mg vs. placebo 4.8 years (mean)	All‐cause mortality ‐ 6‐year mortality rate 14.9% vs. 15.3% for statin vs. placebo, relative risk 0.99; 95% CI, 0.89–1.11, *p* = 0.88	726 participants ≥75 years available for post hoc analysis: HR 1.08 (95% CI 0.85–1.37)
Anglo‐Scandinavian Cardiac Outcomes Trial—Lipid Lowering Arm (ASCOT‐ LLA)^39^, ^47^ 2003	‐ 10,305 adults aged 40–79 ‐ >75 years, *n* = 896 ‐ mixed primary and secondary prevention (10% stroke at baseline) ‐ Ireland, United Kingdom, Scandinavia/Nordic countries	Atorvastatin 10 mg vs. placebo 3.3 years	Composite: non‐fatal myocardial infarction and fatal coronary heart disease ‐ significant reduction in the primary endpoint: 100 vs. 154 events among statin vs. placebo (HR 0.64, 95% CI 0.50–0.83, *p* = 0.0005)	4445 participants ≥65 years (mean age 71): higher baseline stroke rate (14% vs. 7% in <65 years) ‐ significant reduction in MI and fatal CHD for statin vs. placebo: HR 0.63, 95% CI 0.44–0.89, *p* < 0.01
Justification for the Use of Statins in Primary Prevention (JUPITER)^42^, ^48^ 2008	‐ 17,802 men aged ≥50 years and women aged ≥60 years, no upper age limit ‐ >75 years, *n* = 2176 ‐ no prior CVD and elevated C‐reactive protein levels > 2.0 mg/L ‐ multinational	Rosuvastatin 20 mg vs. placebo Planned 5 years, stopped after 1.9 years	Composite: myocardial infarction, stroke, arterial revascularization, hospitalization for unstable angina, or death from cardiovascular causes	Participants >70 years, *n* = 5695 Incident Rate per 100 person‐years for statin vs. placebo: 0.82% vs. 1.36%, HR 0.61, 95% CI 0.43–0.86
Heart Outcomes Prevention Evaluation‐3 (HOPE‐3)^40^, ^42^ 2016	‐ 12,705 men aged ≥55 years and women aged ≥65 years ‐ >75 years, *n* = 1088 ‐ primary prevention, at least 1 CVD risk factor ‐ multinational	Rosuvastatin 10 mg vs. placebo 5.6 years	Composite: cardiovascular death, nonfatal myocardial infarction, or nonfatal stroke.	Participants >70 years, *n* = 5695 Incident Rate per 100 person‐years for statin vs. placebo: 1.25% vs. 1.50%, HR 0.83 (0.64–1.07)

Among the 14,483 participants older than 75 years at randomization included in the Cholesterol Treatment Trialist (CTT) meta‐analysis^35^ who were randomized to a statin versus placebo or more intensive statin therapy vs. placebo or less intensive statin therapy, the absolute event rate was 1051 (4.5%) vs. 1153 (5.0%), RR 0.87 (95% CI 0.77‐0.99). When individuals enrolled in heart failure or dialysis trials were excluded, the absolute event rates were 4.1 and 4.7%, respectively with a RR of 0.82 (95% CI 0.70‐0.95). When the analysis was restricted to the 6,007 participants without prior vascular disease, results were attenuated and no longer statistically significant: 295 (2.7%) vs. 308 (2.8%) events, RR 0.92 (95% CI 0.73‐1.16). This heterogeneity persisted when heart failure and dialysis trials were excluded. A challenge to interpreting these data is the limited sample size and lack of generalizability, including healthy volunteer bias, and trials designed to specifically exclude older adults with complex medical conditions or who do not live independently.^40^, ^41^, ^42^ Leveraging territory‐wide public electronic medical records in Hongkong and a target trial emulation study design, Xu et al. compared cardiovascular outcomes and adverse events among older statin users and non‐users without prior coronary heart disease.^43^ Absolute risk reduction in CVD incidence over 5 years was 1.20% (95% CI 0.57%‐1.82%) among 75 to 84 year old individuals and 4.44% (95% CI 1.40%‐7.48%) among those aged 85 years or older. There was no increase in myopathies or liver dysfunction in either group. Two ongoing randomized trials, the STAREE trial^44^ and the PREVENTABLE Trial^45^ are addressing benefits and risks of statin therapy in this age group prospectively (see Future Directions).

QUESTION 4 **For the population of adults older than 75 years without established ASCVD, how should we weigh potential safety concerns versus benefits of statin therapy?**

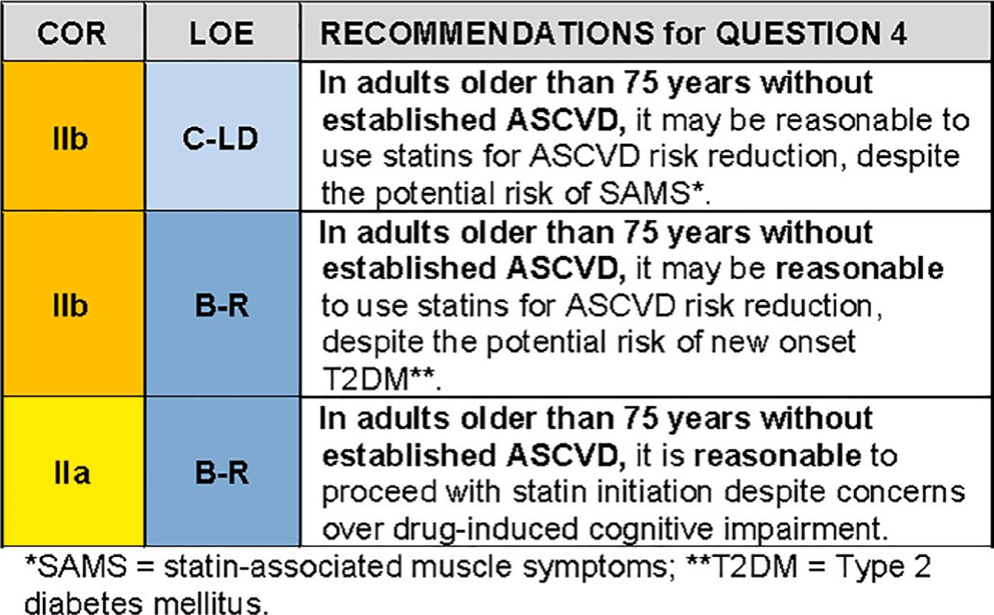

### Synopsis

Adults older than 75 years are intrinsically more susceptible to iatrogenic risks from medications amidst age‐related changes in metabolism, body composition, mitochondrial energetics, and cognition. Sarcopenia, frailty, and insulin resistance are common, along with changes in pharmacokinetics and pharmacodynamics. Therefore, many hypothesize that older adults may be more susceptible to statin‐associated muscle symptoms (SAMS), new onset type 2 diabetes mellitus (T2DM), and cognitive decline if they use statins.

Meta‐analyses of RCTs and recent observational data from patient registries do not indicate any increase in the risk of SAMS among those above age 75 compared to younger individuals.^49^, ^50^ Multiple meta‐analyses suggest that statin therapy leads to a small increase in risk of new onset T2DM. It is estimated that statin therapy is associated with one additional case of T2DM per 255 patients taking statins for 4 years^51^, largely confined to patients with risk factors for T2DM including advanced age.^51^, ^52^ Risk of new onset T2DM is greater with high‐intensity statin regimens than with the moderate‐intensity statins typically used in primary prevention among older adults.^53^ The increase in risk of new onset T2DM associated with statin therapy is outweighed by robust reductions in major cardiovascular events among individuals with T2DM.^51^, ^53^

Analyses of the impact of statin therapy on cognition have significant methodological limitations.^54^ The diagnosis of dementia or cognitive change as an endpoint is rarely measured systematically, and confounders like education level and intelligence are rarely considered. Many studies do not adequately measure longitudinal changes in cognition and types of dementia are generally not distinguished. Few studies use in vivo brain imaging, cerebrospinal fluid analysis or serological biomarkers. While there has been some literature suggesting the possibility that statins may be associated with incident cognitive impairment in older adults, a preponderance of literature indicates neutral or even protective statin‐related cognitive effects.^55^, ^56^, ^57^

### Recommendation‐specific supportive text

1. SAMS: The CTT Collaboration^49^ meta‐analysis of SAMS included 19 double‐blind trials of statin versus placebo (*n* = 123,940) and 4 double‐blind trials of a more intensive versus a less intensive statin regimen (*n* = 30,724). The RR of muscle related events comparing statin and placebo treatment was 1.07 (95% CI 0.95–1.20) among the subset of participants over age 75 years, comparable to RR among those age 65 years or younger (RR 1.03 (95% CI 0.97–1.10)) and those aged older than 65 up to 75 years (RR 1.11 (95% CI 1.04–1.19)). Reports of SAMS are higher in clinical practice than in clinical trials, at least in part because patients with adverse effects during the clinical trial run‐in phase are excluded from participation in the main trial. The Patient and Provider Assessment of Lipid Management registry^50^ enrolled 6717 older individuals (*N* = 1704 (25%) were aged older than 75 years). Among current statin users, older patients were less likely to report any symptoms (41.3% versus 46.6%; *p* = 0.003) or myalgias (27.3% versus 33.3%; *p* < 0.001) than younger patients.
  The SAMSON (Self‐Assessment Method for Statin Side‐Effects Or Nocebo) trial^58^ used a crossover design to assess 60 participants with a history of statin intolerance (85% were SAMS). Although age was not specifically studied, 83% of the population was aged 60 years and older (mean age 65.5±8.6 years). The trial demonstrated that 90% of SAMS were attributable to a nocebo effect, triggered by taking a pill itself, not by the statin in the pill, plausibly because the individual believes that the pill will cause harm. While 26 of 60 participants taking the statin had to stop the medication early due to intolerable side‐effects, so did 23 of 60 taking placebo. However, 6 months after completion of the protocol (i.e., after participants understood their results), 30 of 60 (50%) participants successfully restarted statins, including 10 who had stopped statins during the trial because of side effects. Statin intolerance, and management strategies for SAMS are discussed in other NLA publications.^59^, ^60^

2 New onset T2DM: A post hoc analysis of the JUPITER^52^ trial first demonstrated an increase in new onset T2DM among participants with at least one diabetes‐risk factor randomized to rosuvastatin (HR 1.28, 95% CI 1.07–1.54), but no risk increase among those without risk factors for T2DM. Risks of T2DM were counterbalanced by a 39% reduction in the primary combined endpoint of myocardial infarction, stroke, hospitalization for unstable angina, arterial revascularization, or cardiovascular death (HR 0.61, 95% CI 0.47–0.79) and a nonsignificant 17% reduction in total mortality (HR 0.83, 95% CI 0.64–1.07). In participants with at least one major diabetes risk factor, 134 vascular events or deaths were avoided for every 54 cases of newly diagnosed T2DM. This increase in new onset T2DM risk was subsequently confirmed in three meta‐analyses.^51^, ^53^, ^61^ Pooled data from 13 statin trials showed a 9% increase in new onset T2DM, translating to approximately 1 new case of T2DM for every 255 individuals treated with a statin for 4 years.^51^ The most recent analysis from the Cholesterol Treatment Trialists' Collaboration confirmed the increase in risk of T2DM with low/moderate intensity and with high intensity statin use, but there was no heterogeneity of this risk by age.^53^ In the PROSPER trial of 70–82 year‐old participants, 6.6% of those randomized to pravastatin developed T2DM compared to 5.1% of those allocated to placebo.^38^ A propensity‐matched analysis of Medicare Advantage Prescription Drug claims found a 4.82% cumulative incidence of T2DM over 7 years with an adjusted OR of 1.26 (95% CI 1.12–1.41) among statin users.^62^ Mean age in those with new onset T2DM was 72.1 ± 5.1 years. In an exploratory analysis of PROSPER, Homeostasis Model Assessment of Insulin Resistance (HOMA‐IR) was a powerful predictor of T2DM comparing top and bottom tertiles in models adjusted for baseline characteristics and treatment (HR 4.8, 95% CI 3.14, 7.33), but was not predictive of subsequent ASCVD events or death during 3.2 years of follow‐up. These data suggest that incident T2DM in older patients may not be associated with a worse prognosis.^63^
3 Cognition: In a retrospective analysis of the Effects of Aspirin on All Cause Mortality in the Healthy Old (ASPREE) trial^55^ (*N* = 18,846, median age 74 years, 56.4% women, 31.3% on statin), statin use was not associated with incident dementia, mild cognitive impairment (MCI), or cognitive change over 4.5 years of follow‐up. There was a trend toward an increase in Alzheimer's Disease (HR 1.33; 95% CI: 1.00 to 1.77; *p* = 0.05). Patients in the lowest quartile of baseline cognitive function on statins had higher hazards for dementia and change in episodic memory, a component of cognitive testing most associated with Alzheimer's Disease (P_interaction_ < 0.0001). Samaras et al.^57^ completed a prospective observational study of 1037 older community‐dwelling adults over 6 years comparing statin users (age 78.6 ± 4.8 years) and non‐users (age 79.3 ± 4.8 years). There were no differences in the rate of decline in memory or global cognition between groups. Statin initiation during the observation period was associated with blunting of the rate of memory decline. There were no differences in brain volume changes on magnetic resonance imaging between statin users and non‐users. Adhikari et al.^56^ reported on 1,404,459 participants age ≥ 60 years in a meta‐analysis of 24 studies. In 3 randomized controlled trials (HPS, PROSPER and HOPE‐3) the rate of incident cognitive decline was approximately 20% in the placebo group; no significant association between statin use and adverse cognitive effects were detected.^37^, ^64^, ^65^ Seven out of 10 observational studies showed no association between statins and incident dementia, two showed a similar decline in cognition between statin users and non‐users, and one showed a slower decline with statin use. Smith et al.^66^ found less cognitive decline among older adults (*N* = 443, mean age 73 ± 7.4 years) with MCI who were treated with statins compared to a similar population (*N* = 325, mean age 72.9 ± 7.7 years) who were not treated with statins. Future trials such as PREVENTABLE^45^ and STAREE^44^ will provide further insights on the cognitive effects of statins.

QUESTION 5 **For the population of adults older than 75 years without established ASCVD, how do we assess the expected net benefit of statin therapy?**

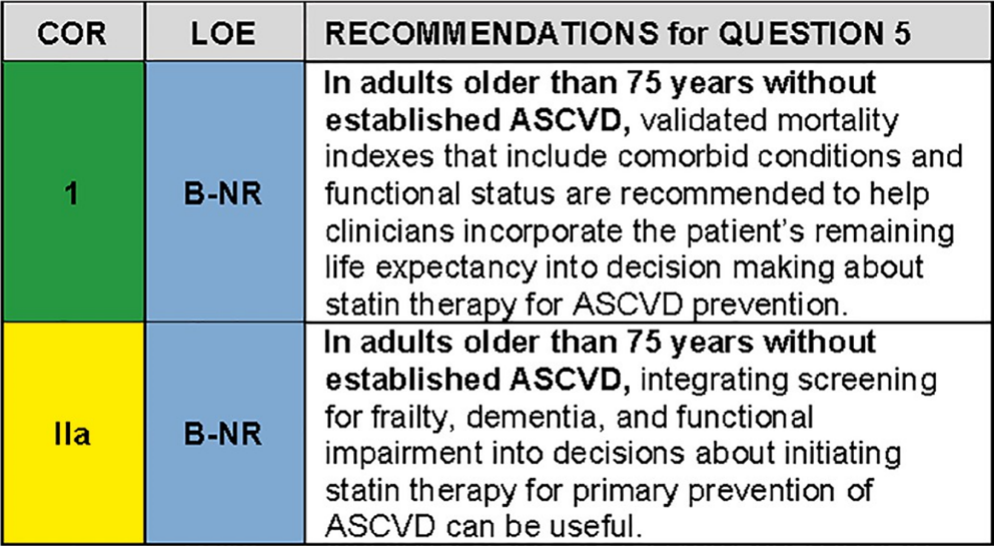

### Synopsis

Comorbidity burden and life expectancy vary widely among older adults of the same age.^67^ There is thus considerable variability in the likelihood that an older adult will benefit from preventive and therapeutic interventions. Any benefit from preventive measures may be attenuated by competing health risks from multiple chronic conditions, and the risk of adverse outcomes may be magnified. Lifetables and validated mortality indexes which consider co‐morbidities are available to allow clinicians to refine their patient's prognosis. Furthermore, as discussed under Question 1, 2, 3, 5, 7, 8, competing risk‐adjusted models can help assess ASCVD risk and potential benefit.

Frailty, a syndrome of biologic aging and vulnerability, ranges in prevalence from 7% in community‐dwelling older adults to over 50% in institutionalized patients.^68^, ^69^ The prevalence of functional limitation is 66% in adults aged 65 years and older (Centers for Disease Control and Prevention. Health, United States, 2020‐2021: Functional Limitation. https://www.cdc.gov/nchs/hus/topics/functional‐limitation.htm. Updated January 16, 2024. Accessed February 21, 2024). In the U.S. in 2022, an estimated 37.2% of people aged 75–84 years and 35.7% of people aged 85 years and older have Alzheimer's dementia and other dementias.^70^ Mild cognitive impairment affects 12‐18% of people aged 60 and older in the United States.^70^ Frailty, cognition and functional impairment should be considered in person‐centered decision making about preventive measures. Numerous tools are available to assess these geriatric conditions at the bedside, and decision aids can be helpful in assessing patient preferences.

### Recommendation‐specifc supportive text

1. There is substantial variability in life expectancy among older adults of the same age, e.g., a healthy 85‐year‐old woman is expected to live an additional 10 years (and would be likely to benefit from primary prevention) while a frail, multimorbid 85‐year‐old woman may have less than 2 years remaining life expectancy and may not stand to benefit.^71^ Clinicians can consider morbidity burden, functional status and frailty to estimate life expectancy.^71^
  If an individual's life expectancy is greater than the time to benefit for a given preventive intervention, the intervention may help and should generally be recommended.^72^ If the life expectancy is shorter than the time to benefit, the intervention is more likely to harm and generally should not be recommended. If the life expectancy is approximately equal to the time to benefit, patient preferences should be the guiding factor in decision making. An online calculator, ePrognosis (https://eprognosis.ucsf.edu/), can help clinicians incorporate evidence‐based information about life expectancy for older adults into decisions about preventive therapies such as statins. For example, for an individual with advanced dementia, the time to benefit of primary prevention statin therapy may exceed life expectancy and the goals of therapy may shift over time from preventing illness and prolonging life to reducing the burden of treatment and maintaining quality of life.^73^
2. For patients with frailty, severe functional impairment or dementia, incorporating patient preferences in decisions about statin therapy is essential for patient‐centered care.^74^ These conditions are associated with physical and functional decline, increased mortality risk,^75^, ^76^ and increased risk of harm from polypharmacy.^77^, ^78^, ^79^ Yet, individuals with frailty, functional impairment and dementia should not automatically be denied statin therapy. Although few studies have included such patients, a retrospective cohort study of 326,981 Veterans aged 75 years and older without ASCVD at baseline found that new statin use was significantly associated with a lower risk of all‐cause and cardiovascular mortality, even at advanced ages (e.g., aged >90 years) and in those with dementia.^80^ This association was evident within 2 years and remained significant in those with frailty.
  However, for some patients with frailty, severe functional impairment or dementia, goals of therapy may shift from preventing illness and prolonging life to reducing the burden of treatment and maintaining quality of life.^73^ Practical tools to assess functional status, frailty and cognition are listed in Table 5. The presence of any of these syndromes may prompt elicitation of patient values and preferences for prevention and medication use (see Question 6, Section 2).

**TABLE 5:** Practical tools to assess geriatric domains of frailty, gait speed, cognition and functional status.

Tool	Description	Time	Online guidance
Frailty: Clinical Frailty Scale^81^	9 categories based on physical function and medical conditions in the past 2 weeks	3 min	https://www.dal.ca/sites/gmr/our‐tools/clinical‐frailty‐scale.html
Chair Stand and Gait Speed^82^, ^83^, ^84^	Focused mobility assessment	2 min	https://www.nejm.org/doi/full/10.1056/NEJMvcm2009406
Cognition: Mini Cog^85^	Brief cognitive screen	3 min	https://mini‐cog.com/download‐the‐mini‐cog‐instrument/
Frailty: CGA‐FI^86^, ^87^	Comprehensive Geriatric Assessment that incorporates medical history, functional, and cognitive status to calculate a frailty index	30 min	https://efrailty.hsl.harvard.edu/tools/CGA‐FI/index.html

QUESTION 6 **In adults older than 75 years without established ASCVD, what strategies should be utilized for initiation, monitoring, and intensification of statin therapy?**

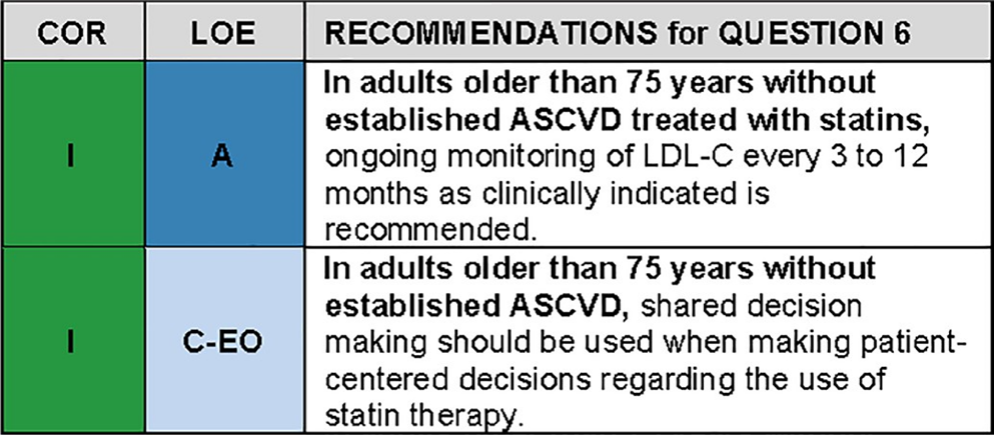

### Synopsis

Safety and efficacy monitoring for statin therapy among older patients follows conventional standards of care. CAC scoring can augment ASCVD risk estimation among older patients without ASCVD and can inform the decision to initiate statin therapy and intensity of statin therapy or inform decisions to discontinue statins. CAC scoring among older patients without ASCVD is addressed in Question 2, Recommendation 3.

Considering the clinical uncertainties described above, incorporating patient priorities and preferences in decisions about statin therapy (including initiation of therapy, continuation of therapy, and monitoring of therapy) is essential to improve patient‐centered preventive care.^74^ Shared decision making involves seeking the patient's participation in clinical decision making, helping the patient explore and compare potential treatment options, assessing the patient's values and preferences, and reaching a decision with the patient. Shared decision making is important for all patients, but it may be particularly useful among older primary prevention patients when there is limited evidence of improved ASCVD outcomes and other patient preferred outcomes with statin therapy.

### Recommendation‐specific supportive text

1. An exploratory analysis of the JUPITER trial compared outcomes in 5695 participants age 70–97 years to those age 50–69 years old.^88^ Within this older subgroup, rosuvastatin reduced major ASCVD events by 39% with favorable point estimates for cardiovascular death and total mortality and greater absolute reductions in major ASCVD events compared to the younger group. Assessment for adverse effects, and measurements of lipid values, liver function tests, and hemoglobin A1C occurred every six months. Serious adverse events were more common in older than younger patients, but similar between rosuvastatin and placebo within each age group. As discussed in Question 4, new onset T2DM was more common in the rosuvastatin group in older patients, but comparable to the increase seen in younger participants. The U.S. Food and Drug Administration removed the recommendation for routine liver function test measurements with statin therapy in 2012. The 2018 AHA/ACC/Multisociety cholesterol guidelines recommend measurement of liver function tests at baseline and if there are signs or symptoms of hepatotoxicity during therapy. The guidelines further recommend a comprehensive evaluation of musculoskeletal symptoms prior to statin treatment because such symptoms are common in the general adult population and especially in older individuals. Serum creatine kinase testing should be performed for patients with severe SAMS or muscle weakness during treatment.^31^ Monitoring of LDL‐C is recommended 4 to 12 weeks after starting therapy or making statin dose adjustments and every 3 to 12 months thereafter as clinically indicated to monitor adherence and safety without distinctions by patient age. Among those with risk factors for the development ofT2DM, monitoring of hemoglobin A1C levels is also recommended.^31^ Clinicians should use these results and shared decision making to manage therapy to achieve patient‐specific therapeutic objectives.
2. The fundamental competent and practical steps of shared decision‐making include: information sharing, patient education, exploring patient preferences, decision support tools, deliberation and discussion, shared decision‐making agreement, and ongoing communication and review.^89^ Given limited evidence of improved ASCVD outcomes with statin therapy in adults age 75 years or older, including these patients in decision making about statin initiation, type of statin and statin intensity, and about continuation or discontinuation of therapy is important to reduce decisional conflict and ensure that therapy is consistent with patient priorities and preferences. Prospective randomized trials that utilized shared decision‐making with or without validated decision tools in younger patients have demonstrated reductions in decisional conflict and increased patient acceptance of statin therapy.^90^, ^91^, ^92^ as has provision of statistics addressing relative risk reduction in ASCVD events.^93^ The Mayo Clinic Statin Choice Decision Aid (https://statindecisionaid.mayoclinic.org/) has been validated in clinical trials^92^, ^94^, ^95^ and is available to facilitate shared decision‐making in clinical practice.
  Older adults with multimorbidity may face multiple preference‐sensitive decisions, and treatment of one condition may exacerbate another condition, making it impractical to use decision aids for each choice. A better approach to eliciting preferences among older adults with multimorbidity may be to ask patients to rank a set of universal health outcomes such as living as long as possible, preserving function, and alleviating pain and other symptoms.^96^, ^97^, ^98^ The clinician and patient can then consider these factors within the shared decision‐making process. Statin therapy could be considered in terms of how likely it is to help the patient achieve their most‐desired outcome or avoid their least‐desired outcome. Understanding a patient's priorities can help to inform the overall approach to medications, including statins.^98^

QUESTION 7 **In adults older than 75 years without established ASCVD, what are the best practices for statin deprescribing?**

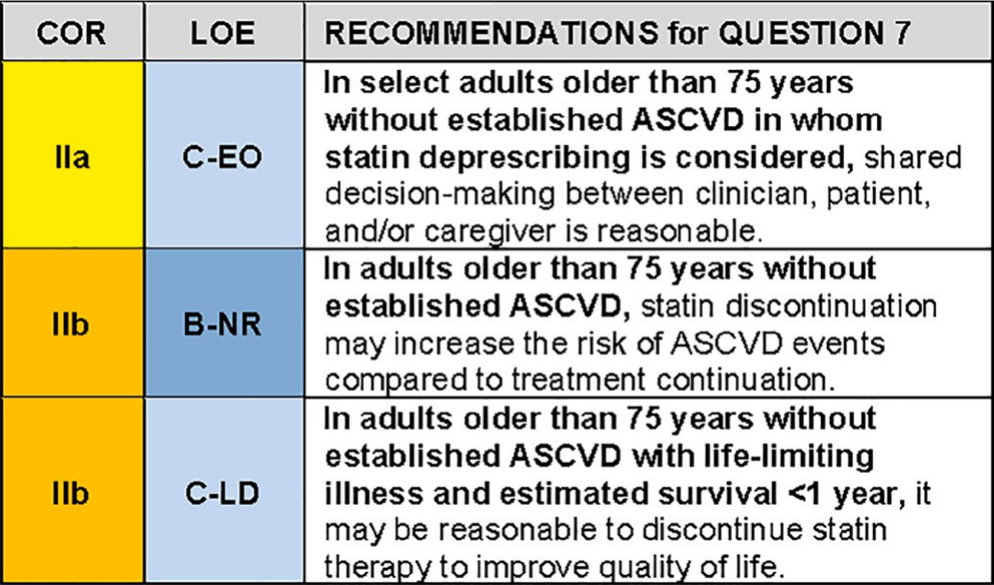

### Synopsis

Deprescribing medications is an important part of the prescribing continuum and an essential component of high‐quality care for older adults with multiple comorbidities, geriatric syndromes and polypharmacy who are more susceptible to medication‐related harms. Benefits of deprescribing statins (e.g., taking fewer medications, avoiding interactions with other medications such as antibiotics or antifungal agents that are metabolized through the same pathway as the statin, avoiding adverse effects in general) must be balanced against the potential for harm, such as an increase in ASCVD events after statin discontinuation. General principles of deprescribing include viewing deprescribing as a routine part of care designed to improve patient well‐being (rather than withdrawal of appropriate care) and shared decision‐making with the patient/caregiver.^99^ Three large cohort studies provide evidence for clinicians and patients regarding the risks and benefits of statin discontinuation on cardiovascular events in older adults without a history of cardiovascular disease who are taking statins (Table 6).^100^, ^101^, ^102^ One small prospective trial found benefits of deprescribing among older adults near the end of life.^103^

**TABLE 6:** Studies of Statin Deprescribing.

Reference	Study Design	Patient Characteristics	Outcomes/Results
Giral et al.^100^	French national healthcare database study Marginal structural model, adjusting for baseline and time‐varying covariates (cardiovascular drug use, comorbidities, and frailty indicators) Statin discontinuation vs. continuation Mean 2.4 years follow‐up	*N* = 120,173 14% discontinued statin Age: 75 years Primary prevention Previously adherent to statins for 2 years	Admission for CV event ◦ 5396 total patients admitted for CV event, crude incidence rate of 2.1/100 patient‐years ◦ Statin discontinuation: adjusted HR 1.33 (95% CI 1.18–1.50). Admission for coronary and cerebrovascular events: ◦ Statin discontinuation: adjusted HR 1.46 (95% CI 1.21–1.75) and 1.26 (95% CI 1.05–1.51), respectively After 4 years of follow‐up (all patients had aged to 79 years): adjusted cumulative incidence rate of CV events 10.1% with statin discontinuation compared to 7.6% for statin continuation. Analysis based on baseline characteristics (sex, diabetes, antihypertensive drug use, comorbidities, frailty, intensity of statin therapy) showed no significant heterogeneity.
Thompson et al.^101^	Denmark Statin discontinuation vs. continuation Mean 5.5 years follow‐up in the primary prevention cohort	*N* = 67,418 30% discontinued statin in the primary prevention cohort Age: 75+ years (median 79 years and 66% female in PP cohort) Primary (41%) and secondary prevention (59%) Treated and adherent for at least 5 years to a statin	MACE in the primary prevention cohort ◦ Statin discontinuation: crude incidence of MACE 33/1000 person‐years compared to 24/1000 person‐years with continuation group ◦ Crude and weighted difference in the incidence rate of MACE: 9/1000 patient‐years (95% CI, 5–12 per 1000 person‐years), corresponding to 1 excess MACE per 112 discontinuers per year ◦ With discontinuation, HR for MACE occurrence 1.32, 95% CI 1.18–1.48 ◦ With discontinuation, HR for myocardial infarction occurrence 1.37, 95% CI 1.11–1.70 With discontinuation, HR for ischemic stroke/transient ischemic attack occurrence 1.33, 95% CI 1.14–1.54
Rea et al.^102^	Italy Statin discontinuation vs. continuation Evaluation group: 4010 who discontinued statins were propensity‐score matched with a comparator group from the larger group who maintained statin use Mean 20‐month follow‐up	*N* = 29,047 20% discontinued statin Age: 65+ years (mean 76 years) Primary (approx. 72%) and secondary prevention Taking statins continuously for 15 months	Statin discontinuation in overall cohort: ◦ Hospital admission for cerebrovascular disease: Incidence rate 35.8/1000 person‐years vs. 31.2/1000 person‐years with continuation; RR 1.15, 95% CI 0.95–1.38 ◦ Hospital admission for ischemic heart disease: Incidence rate 69.7 per 1000 person‐years vs. 64.6 per 1000 person‐years with continuation; RR 1.08, 95% CI 0.94–1.23 ◦ Composite CV outcome: RR 1.14, 95% CI 1.03–1.26 Statin discontinuation in the primary prevention cohort: Composite CV outcome: HR 1.14, 95% CI 1.00–1.30
Kutner et al.^103^	Prospective, randomized, parallel trial Statin discontinuation vs. continuation Median follow‐up 18 months	*N* = 381 Primary (42%) and secondary (58%) prevention Life expectancy < 1 year Recent deterioration in functional status No recent active CV disease Mean age 74.1 years 69% had taken statins > 5 years 1/3 enrolled in hospice 50% had cancer 22% cognitively impaired	Death at 60 days: Discontinuation vs. continuation 23.8% vs. 20.3%; *p* = 0.36 24 (6.3%) of the patients experienced their first CV‐related event during the study period without significant difference between groups (*p* = 0.64) Statin discontinuation reduced medication‐related costs ($3.37 per day; calculated based on the 2012 national average retail price of statins) Statin discontinuation improved total quality of life (mean McGill QOL score, 7.07 vs. 6.74; *p* = 0.03 based on area under the curve up to 20 weeks) Statin discontinuation compared to statin continuation, did not reduce physical symptoms, performance status, or statin‐associated symptoms

### Recommendation‐specific supportive text

1. Clinical decision making and communication for deprescribing statins can be complex regardless of the patient's medical conditions and frailty. It is prudent for clinicians to have thoughtful conversations with their patients and their caregivers regarding the potential benefits and risks of statin continuation and deprescribing in older age. Some clinicians may need to acknowledge and overcome their own uncertainty about stopping statins if their patients are interested in discontinuation. A study of 180 acutely ill Australian older adults found that 95% were willing to stop their statin, predominantly over concerns for adverse effects.^104^ When discussing statin discontinuation through shared decision‐making, clinicians may also need to alter their communication about deprescribing based on their patient's preferences. A survey of 90 Danish general practitioners identified that the most important topic clinicians wanted to discuss related to stopping statins was goals of therapy, while adverse effects were considered less important.^105^ However, a U.S. study of 835 older adults found that most patients preferred phrasing focused on the risk of adverse effects when discussing deprescribing a statin.^106^ Although existing data suggest the risk of statin‐related adverse effects is low, older adults with frailty, severe functional or cognitive impairment, or polypharmacy have not been well‐represented in studies that assessed adverse effects. Adverse effects, even mild ones, may play a greater role in driving decisions about statin therapy for such patients, and observational data suggest that nonspecific symptoms such as fatigue and dizziness are associated with the total number of medications as well as with individual medications.^107^ Another small qualitative study of clinicians, caregivers and older adults with dementia found that patients and caregivers preferred clinicians to explain that aging and comorbid conditions may shift the balance of benefits and risks of medications such as statins. Examples of such phrasing are “Our bodies change over time and certain medicines may no longer be needed” and “These medications take years to have an effect and I think that we should focus on what can help you right now”.^108^
2. Three observational cohort studies of older adults taking statins for primary prevention in France, Denmark and Italy (aggregate *N* > 200,000 individuals) reported an increased risk of cardiovascular events over a subsequent 2–5 year period among patients who stopped their statin, compared to patients who continued (Table 6).^100^, ^101^, ^102^ Adjusted HRs suggested an approximately 30% increase in admissions for ASCVD events during follow‐up among those who discontinued statins. The reasons for statin discontinuation were unknown. While investigators adjusted for baseline characteristics or performed propensity‐matching prior to data analysis, results may in part reflect residual unmeasured or unknown confounding. It is also unknown whether these estimates translate to the U.S. population. Nevertheless, these studies provide risk estimates for statin discontinuation that can be incorporated into shared decision‐making conversations.
3. Deprescribing may provide benefits for patients near the end of their life. Kutner et al. evaluated 60‐day survival after statin deprescribing in an unblinded randomized, parallel group non‐inferiority trial of 381 individuals with life‐limiting illness and an estimated survival of < 1 year; 36% of patients were enrolled in hospice. There were no differences in 60‐day survival or cardiovascular events between the 2 groups. Quality of life was improved and medication cost (using 2012 retail pricing) lowered among those who discontinued their statin.^103^ These results indicate that it may be reasonable to discontinue statin therapy in patients with limited life expectancy due to co‐morbid conditions. Further research would be helpful to guide deprescribing discussions in this population.

QUESTION 8 **For the population of adults older than 75 years without established ASCVD, when should non‐statin therapies be considered for ASCVD risk reduction?**

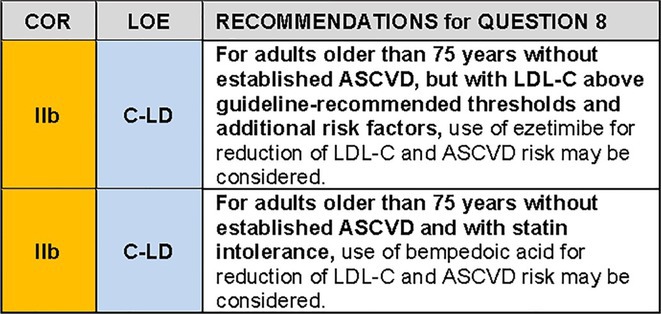

### Synopsis

Due to a lack of randomized controlled trials, non‐statin therapy for adults older than 75 years without prevalent ASCVD was not addressed in recent US or ESC guidelines of cholesterol management.^31^, ^109^, ^110^, ^111^ Ezetimibe as monotherapy has been evaluated since in a single randomized controlled open label trial, EWTOPIA 75 (Ezetimibe Lipid‐Lowering Trial on Prevention of Atherosclerotic Cardiovascular Disease in 75 or Older) which showed a 2.6% absolute and 34% relative reduction in risk of MACE in the ezetimibe group.^112^ The combination of statin and ezetimibe has not been evaluated in outcomes trials among adults older than 75 years without ASCVD. Among adults older than 75 years in whom a decision is made to use LDL‐C lowering therapy for the primary prevention of ASCVD, statins are preferred given the available evidence, but ezetimibe therapy may be considered as an alternative if statin therapy is not tolerated, deemed inadvisable, or based on patient preference.

Limited data on bempedoic acid among statin‐intolerant high‐risk primary prevention patients are available from a subgroup analysis of the CLEAR Trial (Bempedoic Acid and Cardiovascular Outcomes in Statin‐Intolerant Patients). Bempedoic acid reduced the primary endpoint of major adverse cardiovascular events by 30% (absolute risk reduction 2.3% (5.3% vs. 7.6%)). The mean age among primary prevention patients was 68 years. In the overall trial, 15% of participants were aged 75 years or older, but data specific to this subgroup were not provided. There was no interaction of treatment by age.^113^ Thus, among statin intolerant individuals older than 75 years in whom a decision is made to lower LDL‐C to reduce ASCVD risk, bempedoic acid may be considered.

Secondary prevention trials with both evolocumab and alirocumab suggest similar benefits and safety of PCSK9 inhibition in older (≥65 years of age) and younger individuals.^114^, ^115^ To date, there are no large randomized controlled outcomes trials using PCSK9 inhibition for primary prevention of ASCVD. Current data are not sufficient to make a recommendation for the treatment of adults aged 75 or older without ASCVD.

**FIGURE 3:**
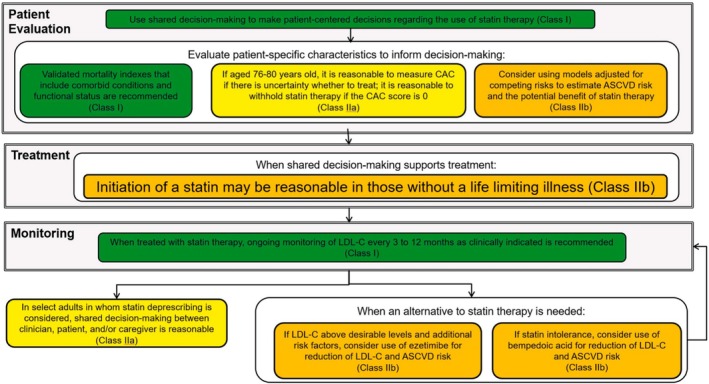
Managing hypercholesterolemia in primary prevention patients older than 75 years, with LDL‐C 70–189 mg/dL. Colors correspond to class of recommendation in Figure 1. ASCVD, atherosclerotic cardiovascular disease; CAC, coronary artery calcium; LDL‐C, low density lipoprotein cholesterol.

Figure 3 summarizes some key steps in managing hypercholesterolemia among this population.

### Recommendation specific text

1. The EWTOPIA trial^112^ randomized 3796 Japanese patients aged 75 years or older without coronary artery disease to ezetimibe or usual care and followed them for a median of 4.1 years; 19% of patients were ≥ 85 years old, mean age was 80.6 years. Patients had an LDL‐C ≥ 140 mg/dL off lipid‐lowering therapy for at least 4 weeks and had at least one of the following: diabetes (present in 25%), hypertension, smoking, low HDL‐C, hypertriglyceridemia, or a history of stroke (present in 7%) or PAD (present in 3%). The mean baseline LDL‐C level was approximately 162 mg/dL. Due to loss of follow‐up, withdrawn consent, and trial irregularities, only 1716 ezetimibe patients and 1695 usual care patients were analyzed. The level of LDL‐C at 1 year was 144.1 mg/dL among usual care and 126.1 mg/dL in the ezetimibe group, translating to an incremental LDL‐C reduction of approximately 18 mg/dL in the ezetimibe group. The primary outcome, a composite of sudden cardiac death, fatal/nonfatal myocardial infarction, coronary revascularization, or fatal/non‐fatal stroke, was reduced by 34% (133 events in the control group [7.8%], 89 events in the ezetimibe group [5.2%], HR 0.66, 95% CI 0.50–0.86, *p* = 0.002). It is of note that the primary outcome was significantly reduced among patients without a history of stroke or PAD and there was no significant interaction by presence or absence of diabetes, chronic kidney disease or within any other pre‐defined subgroup. Regarding the secondary outcomes, the incidences of composite cardiac events (HR, 0.60; 95% CI, 0.37–0.98; *p* = 0.039) and coronary revascularization (HR, 0.38; 95% CI, 0.18–0.79; *p* = 0.007) were lower in the ezetimibe group than in the control group; however, there was no difference in the incidence of stroke, all‐cause mortality, or adverse events between trial groups.
2. In the CLEAR Outcomes trial (Bempedoic Acid and Cardiovascular Outcomes in Statin‐Intolerant Patients) enrolled both secondary prevention patients and high‐risk primary prevention patients.^113^ In the overall cohort, 15% of participants were 75 years or older. Primary prevention patients were on average older than the overall trial population (mean 68 years vs. 65.5 years) and had a higher prevalence of diabetes, but the proportion who were older than 75 years has not been published.^116^ Approximately 20% of primary prevention participants were taking very low dose statins at entry to the trial. There was no interaction of treatment by age for the primary endpoint (major adverse cardiovascular events) in the overall trial analysis, but confidence intervals were wide.^113^ Risk reduction for the primary endpoint was numerically greater in the primary prevention cohort (HR 0.70 [95% CI, 0.55–0.89]) than in the secondary prevention cohort (HR 0.91 [0.82 to 1.01]), p for interaction 0.03.^113^, ^116^ Absolute risk reduction among primary prevention patients was 2.3% (5.3% vs. 7.6%). Outcomes and adverse events among the primary prevention cohort have not been reported for those older than 75 years.

## Future directions

Several ongoing randomized trials will help close the knowledge and evidence gap for statin benefit in older adults without a history of ASCVD and will provide guidance on selection of older patients for deprescribing statins.

### Randomized Controlled Outcomes Trials of Statins in Older Patients Without ASCVD

The Statin Therapy for Reducing Events in the Elderly (STAREE) trial enrolled approximately 10,000 Australians aged 70 years and older to evaluate the effects of atorvastatin 40 mg daily compared to placebo in patients free of ASCVD, diabetes, and dementia.^44^ Patient‐oriented outcomes being assessed over an expected six years of follow‐up include: disability‐free survival, major cardiovascular events, dementia, stroke, heart failure, cancer, hospitalization/institutionalization, and quality of life. The study is expected to be complete in 12/2025 (clinicaltrials.gov, accessed 8/14/2024). This will be the first randomized controlled trial exclusively in older adults without a history of ASCVD.

A second randomized controlled trial, PRagmatic EValuation of evENTs And Benefits of Lipid‐lowering in oldEr Adults (PREVENTABLE), is currently enrolling 20,000 community‐dwelling US adults aged at least 75 years free of ASCVD, dementia, and disability.^45^ Similar to STAREE, PREVENTABLE will evaluate the effects of atorvastatin 40 mg daily vs. placebo for 5 years (estimated median follow‐up 3.8 years) and for similar patient‐oriented outcomes (e.g., prevention of MCI and dementia, disability‐free survival, incident ASCVD events, hospitalization). Secondary outcomes include physical performance, frailty, heart failure, and quality of life. Key differences in PREVENTABLE compared to STAREE include older age or participants and potential for enrollment of higher numbers of diverse individuals.^45^ PREVENTABLE is expected to complete in 12/2026 (clinicaltrials.gov, accessed 8/14/2024).

### Randomized Controlled Trials of Statin Deprescribing in Older Patients

Two large prospective deprescribing studies will help inform clinicians and patients about the benefits and risks of deprescribing statins in older adults without a history of ASCVD. The SITE study^117^ enrolled 1230 French participants aged 75 years and older who were taking statins for primary prevention of ASCVD. Patients were randomized to continue or discontinue their statin, with a follow‐up of 3 years. The study was completed in January 2023 and will report on outcomes of incremental cost per quality‐adjusted life year gained, overall mortality, quality of life, cardiovascular events, T2DM, and cognitive disorders. The STREAM study (https://clinicaltrials.gov/study/NCT05178420) in Switzerland is actively enrolling 1800 participants and is expected to be complete in November 2026. The study is a non‐inferiority clinical trial of adults aged 70 years and older who are taking a statin for primary prevention of ASCVD and are randomized to either continue or discontinue their statin with a follow‐up of a mean of 24 months. The major outcomes of the study will be all‐cause death and major non‐fatal cardiovascular events.

## Conclusion

The STAREE and PREVENTABLE trials will shed light on the benefits of statin initiation in adults over age 70 years. The SITE and STREAM studies will be the first large prospective trials to inform the benefits and risks of discontinuing statins later in life. Future trials should include more diverse study  articipants to better represent the diverse patient populations treated with statins in the US and globally. In addition, better risk stratification methods in older individuals using improved risk equations derived from diverse cohorts, new imaging modalities or new biomarkers may further enhance ASCVD prediction allowing better targeting of those at greatest risk. While we await further evidence, in those who do not have a life limiting illness, primary prevention therapy with a statin can be considered as part of shared decision‐making.

### Evidence

The content of this manuscript is supported by citations, which are listed in the References section.

## Ethics Statement

This submission did not involve human test participants or volunteers.

## Peer Review

This Expert Clinical Consensus underwent peer review. Non‐author editors of the journal and leadership from the National Lipid Association and American Geriatrics Society managed peer review.

## Declaration of Artificial Intelligence and AI‐assisted Technologies in the Writing Process

Artificial intelligence was not used in the writing of the text nor creation of its figures.

## Disclaimers and Limitations

This Expert Clinical Consensus is intended to be an educational tool that incorporates the current medical science and the clinical experiences of lipid specialists, geriatricians, and pharmacists. The intent is to facilitate and improve the clinical care and management of patients. This Expert Clinical Consensus should not be interpreted as “rules” and/ or directives regarding the medical care of an individual patient. The decision regarding the optimal care of the patient is best facilitated with a patient‐centered approach, managed by the clinician tasked with directing an individual treatment plan. In areas regarding inconclusive or insufficient scientific evidence, the authors used their professional judgment. This statement is not a substitute for maintaining awareness of emerging science. Finally, decisions by clinicians and healthcare professionals to apply the principles in this Expert Clinical Consensus are best made by considering local resources, individual patient circumstances, patient agreement, and knowledge of federal, state, and local laws and guidance.

## Updating

This Expert Clinical Consensus may require future updates. The timing of such an update will be determined by the National Lipid Association and American Geriatrics Society.

## CRediT Authorship Contribution Statement

**Vera Bittner:** Writing – review & editing, Writing – original draft, Methodology, Conceptualization. **Sunny A. Linnebur:** Writing – review & editing, Writing – original draft, Methodology, Conceptualization. **Dave L. Dixon:** Writing – review & editing, Writing – original draft, Methodology. **Daniel E. Forman:** Writing – review & editing, Writing – original draft, Methodology. **Ariel R. Green:** Writing – review & editing, Writing – original draft, Methodology. **Terry A. Jacobson:** Writing – review & editing, Writing – original draft, Methodology. **Ariela R. Orkaby:** Writing – review & editing, Writing – original draft, Methodology. **Joseph J. Saseen:** Writing – review & editing, Writing – original draft, Methodology. **Salim S. Virani:** Writing – review & editing, Writing – original draft, Methodology.

## Declaration of Competing Interest

VB received research support from Amgen, DalCor, Esperion, Novartis, Sanofi and from the National Institute on Aging via subcontract from Atrium Health/Wake Forest University; is a data safety monitoring board member for Eli Lilly and Verve Therapeutics; and attended an advisory board for New Amsterdam Pharma. SAL has no disclosures. DLD received research funding from Boehringer Ingelheim. DEF received research funding from the National Institute on Aging, Veterans Health Administration, and the Patient‐Centered Outcomes Research Institute. ARG received research support from the National Institute on Aging and the National Institute on Aging IMbedded Pragmatic Alzheimer’s disease and AD‐Related Dementias Clinical Trials Collaboratory. TAJ has no disclosures. ARO received funding from the National Institute on Aging and the Department of Veterans Affairs. JJS was a data safety monitoring board member for Amgen. SSV received research support from National Institutes of Health, United Kingdom National Institutes for Health and Care Research, Department of Veterans Affairs, Tahir and Jooma Family, and Asharia Family.
